# Supplementary material for: Comprehensive Annotation and Functional Exploration of MicroRNAs in Lettuce
Source: Front Plant Sci. 2021 Dec 24;12:781836. doi: 10.3389/fpls.2021.781836 (PMC8739914; doi:10.3389/fpls.2021.781836)
Supplement: Supplementary file 3 [file Data_Sheet_9.ZIP › GSM2230753.plot/Lsa-miRN1699a_Lsat_1_v5_gn_7_54700.1_4466_TPlot.pdf]

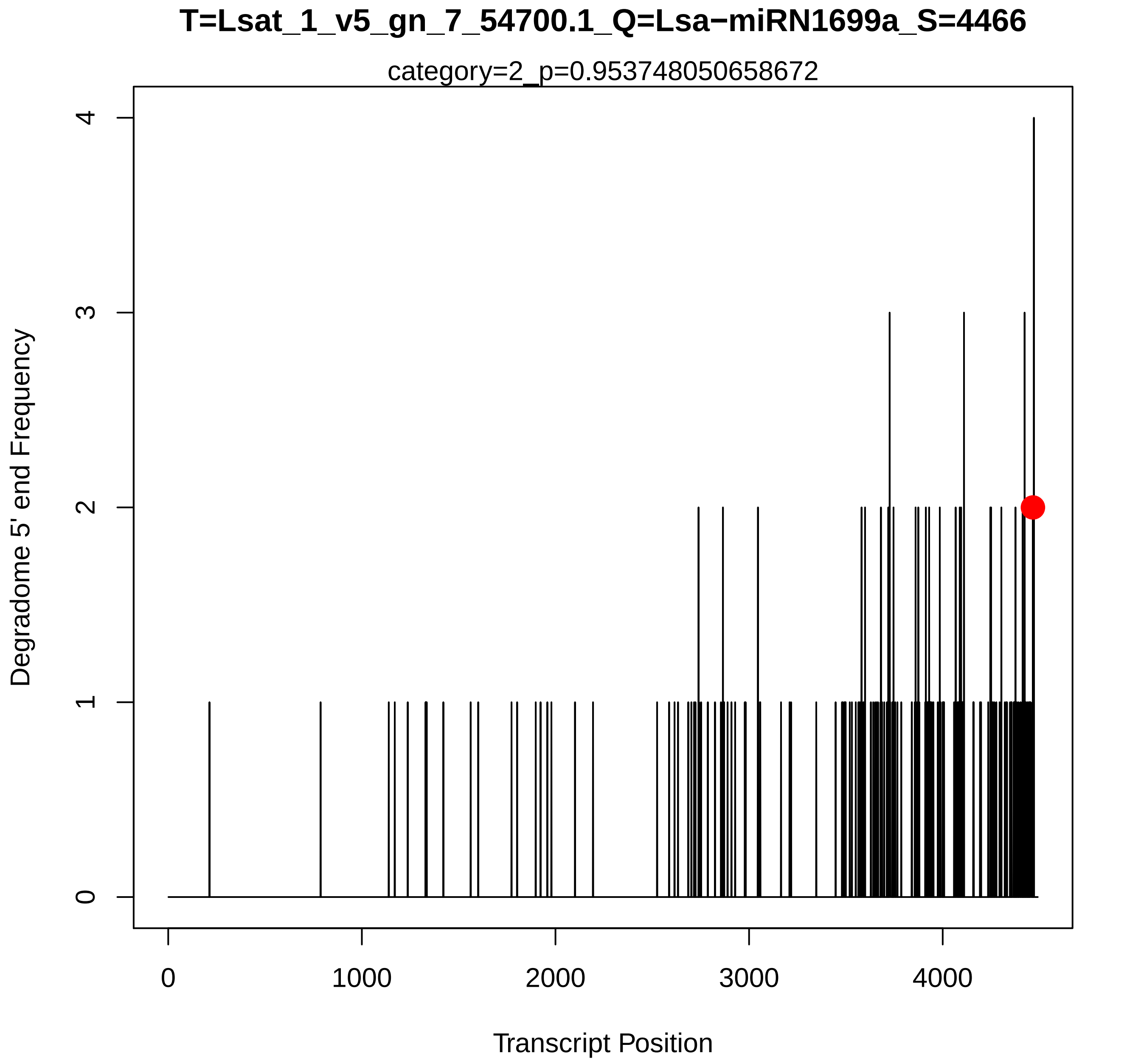
T=Lsat_1_v5_gn_7_54700.1_Q=Lsa−miRN1699a_S=4466
category=2_p=0.953748050658672
Degradome 5' end Frequency
0
1
2
3
4
0
1000
2000
3000
4000
Transcript Position
